# Author response to: Comment on: Double faecal immunochemical testing in patients with symptoms suspicious of colorectal cancer

**DOI:** 10.1093/bjs/znad121

**Published:** 2023-06-01

**Authors:** Adam D Gerrard, Malcolm G Dunlop, Farhat V N Din

**Affiliations:** Cancer Research UK Scotland Centre, Institute of Genetics and Cancer, University of Edinburgh, Edinburgh, UK; Department of Colorectal Surgery, Western General Hospital, Edinburgh, Scotland; Cancer Research UK Scotland Centre, Institute of Genetics and Cancer, University of Edinburgh, Edinburgh, UK; UK Colon Cancer Genetics Group, Medical Research Council Human Genetics Unit, Medical Research Council Institute of Genetics & Cancer, Western General Hospital, The University of Edinburgh, Edinburgh, UK; Cancer Research UK Scotland Centre, Institute of Genetics and Cancer, University of Edinburgh, Edinburgh, UK; Department of Colorectal Surgery, Western General Hospital, Edinburgh, Scotland

*Dear Editor*

Chong *et al*.^1^ asked some questions about our recent paper ‘Double faecal immunochemical testing in patients with symptoms suspicious of colorectal cancer’^2^, after discussion at ‘CRAMSURG’ journal club. When we commenced this study, the utility of the faecal immunochemical test (FIT) in high-risk symptomatic patients was unknown. We aimed to use the FIT for all high-risk referrals. Study patients were triaged to endoluminal investigation regardless of the FIT result, which reflected the real-world use of the FIT in the referred population. Importantly, to the best of our knowledge, this was the first study to systematically assess the false-negative rate in this population. All patients with anaemia (haemoglobin less than 135 g/l in men and less than 120 g/l in women) were included, as we observed an increased colorectal cancer (CRC) risk, regardless of iron deficiency. Processing of double FITs in practice required prior agreement with necessary stakeholders (NHS health board, biochemistry labs etc.), leading to commencement in March 2020. The study intervals differed, initially to ensure similar recruitment numbers, given our expectation for reduced participation in the case of two tests, and latterly as interim results showed that the double-FIT pathway was directly impacting on patient management and therefore we could not continue longer with the same methodology. The distribution of symptoms reported by recruits did change during the COVID-19 lockdown interval. Similarly, routine blood tests for chronic disease in primary care that may have detected incidental anaemia were not being performed. However, patient demographics, FIT positivity, and advanced colorectal neoplasia rates were wholly comparable between the two groups (Table 1)^2^. CT colonography (CTC) was used due to reduced endoscopy during COVID-19 response measures. Whilst this may have missed cases of inflammatory bowel disease, it was reassuring that no CRC was diagnosed within the minimum 1 year of follow-up after CTC. There was no difference in FIT detection rates for left- and right-sided CRC. We agree that their likelihood ratio calculations suggest that double testing, if negative, may reassure against serious pathology.
